# The Perils of Adapting to Dose Errors in Radiation Therapy

**DOI:** 10.1371/journal.pone.0125335

**Published:** 2015-05-05

**Authors:** Velibor V. Mišić, Timothy C. Y. Chan

**Affiliations:** 1 Operations Research Center, Massachusetts Institute of Technology, Cambridge, Massachusetts, United States of America; 2 Department of Mechanical and Industrial Engineering, University of Toronto, Toronto, Ontario, Canada; University of Nebraska Medical Center, UNITED STATES

## Abstract

We consider adaptive robust methods for lung cancer that are also *dose-reactive*, wherein the treatment is modified after each treatment session to account for the dose delivered in prior treatment sessions. Such methods are of interest because they potentially allow for errors in the delivered dose to be corrected as the treatment progresses, thereby ensuring that the tumor receives a sufficient dose at the end of the treatment. We show through a computational study with real lung cancer patient data that while dose reaction is beneficial with respect to the final dose distribution, it may lead to exaggerated daily underdose and overdose relative to non-reactive methods that grows as the treatment progresses. However, by combining dose reaction with a mechanism for updating an estimate of the uncertainty, the magnitude of this growth can be mitigated substantially. The key finding of this paper is that reacting to dose errors – an adaptation strategy that is both simple and intuitively appealing – may backfire and lead to treatments that are clinically unacceptable.

## Introduction

The treatment of lung cancer using intensity-modulated radiation therapy (IMRT) is challenging in part due to the uncertainty that arises from breathing motion during delivery and potential interfractional changes in the patient’s breathing. In [1], the authors proposed the first method for lung cancer IMRT that combines robust optimization (RO) [2, 3] and adaptive radiation therapy (ART) [4]. In this adaptive robust method, the treatment planner specifies an uncertainty set of breathing patterns before the start of treatment. Prior to each fraction, an optimization problem is solved to determine the corresponding fluence map. During or after each fraction, the patient’s breathing pattern is measured, and is used to update the uncertainty set for the next fraction. The process is then repeated until the end of the treatment. They showed that this adaptive robust method results in further improvements in both cumulative tumor coverage and healthy tissue dose relative to the non-adaptive robust approach of [3], which is already capable of providing tumor coverage comparable to a margin treatment but with significantly less dose to the healthy tissue. More recently, [5] showed that this approach is also able to account for drift in the patient’s breathing pattern.

In this paper, we study a different paradigm for treatment planning, which we refer to as *dose reaction*. A dose-reactive method is a treatment planning method in which the target dose for the upcoming fraction is updated based on the dose delivered to date and the treatment is then re-optimized prior to the next fraction. In this way, the method is potentially able to correct errors in dose as the treatment progresses. However, due to the regular modification of the target dose, the daily doses that are delivered over the treatment may vary and deviate significantly from the *daily* prescribed dose. This is important clinically because the effect of deviating from the daily prescribed dose in a given fraction is compounded as the treatment progresses. This can potentially result in treatment failure, even when the final dose meets the prescribed requirements [6].

The purpose of this paper is *not* to develop a new dose-reactive method and to present the merits of this method relative to existing methods for lung cancer IMRT. Rather, our objective is to demonstrate the surprising negative finding that combining dose reaction with an existing adaptive robust framework is *not* necessarily advantageous. We make the following contributions:

1. We show computationally using real patient data that although dose reaction leads to improvements in the final cumulative dose, it may result in explosive growth in the daily underdose and daily overdose over the course of the treatment, and in increased spatial heterogeneity in the daily dose distribution.
2. We show that this growth in daily underdose and overdose in the presence of the dose-reactive element persists when robustness is used without uncertainty set adaptation, but can be substantially mitigated through regular adaptation of the uncertainty set.
3. We prove that uncertainty set adaptation without dose reaction guarantees diminishing daily underdose and overdose asymptotically and provide theoretical justification for why dose reaction will generally lead to growing daily underdose and overdose.

Since [4], there has been increasing interest in ART methods (e.g., [7], [8], [9], [10], [11], [12]). Dose-reactive methods for fractionated radiation therapy, outside of lung cancer IMRT, constitute a major subset of the ART literature (e.g., [13], [14], [15], [16], [17] and [18]). However, few studies in dose reaction account for the daily dose. One example is [19], which builds on [20]. In [19], the authors consider an iterative IMRT optimization approach that adapts to interfraction changes in the tumor geometry and aims to satisfy both per-fraction and cumulative dose constraints. Their approach differs from ours in that it optimizes the number of fractions, which results in an integer programming problem, whereas ours keeps the number of fractions fixed and leads to a linear programming problem. Also, our approach considers both intrafraction and interfraction uncertainty through a robust optimization model with adaptation.

Another closely related paper is that of [21], who proposes a framework where, in each fraction *i* of an *n*-fraction treatment, one delivers 1/(*n* − *i* + 1) of the residual dose distribution—the difference of the dose delivered so far and the target dose distribution. Our work builds on [21] in three key aspects. First, our method uses RO, so that the fluence map delivered in each fraction is designed to deliver the target dose under many instances of the uncertainty; [21] assumes the tumor to be in a single position. Second, our method updates an estimate of the uncertainty by updating an uncertainty set, whereas [21] assumes the tumor to be in the same position from fraction to fraction. As stated in contribution #2, these two differences lead to important insights: the explosive growth in daily underdose and overdose that is alluded to in [21] occurs even in the presence of robustness, but it is possible to reduce its severity through uncertainty set adaptation. Lastly, the results of [21] do not actually show how the daily underdose and overdose increase, and their results pertain only to a stylized one-dimensional model.

## Method

We begin by describing the two elements of our framework: a previously developed adaptive robust optimization model (the “Robust optimization model with adaptation” subsection) and the new dose reaction component (the “Dose reaction” subsection). For the benefit of the reader, Table 1 provides a summary of all of the mathematical notation used here and in the later section titled “Theoretical insight into daily dose performance” that presents our theoretical results.

**Table 1:** Summary of mathematical notation.

Symbol	Description
*x*	Breathing motion state (e.g., full inhale)
*X*	Set of breathing motion state
*p*(*x*)	Probability of breathing motion state *x*
**p**	Breathing motion probability mass function (PMF); **p** = (*p*(*x*))_*x* ∈ *X*_
ℓ(*x*)	Lower bound on *p*(*x*)
***ℓ***	Vector of lower bounds; ***ℓ*** = (ℓ(*x*))_*x* ∈ *X*_
*u*(*x*)	Upper bound on *p*(*x*)
**u**	Vector of upper bounds; **u** = (*u*(*x*))_*x* ∈ *X*_
*P*	Uncertainty set (set of possible breathing motion PMFs)
𝓟	Set of all possible PMFs (the (|*X*| − 1)-dimensional unit simplex)
*v*	Voxel index
𝓥	Set of all voxels
𝓣	Set of tumor voxels
*b*	Beamlet index
*β*	Set of all beamlet indices
*w*_*b*_	Intensity of beamlet *b*
**w**	Beamlet intensity vector; **w** = (*w*_*b*_)_*b* ∈ *β*_
Δ_*v*, *x*, *b*_	Dose deposition coefficient; dose delivered to voxel *v* by beamlet *b* at unit intensity when the motion state is *x*
*θ*_*v*_	Minimum cumulative prescription dose for voxel *v*
*γ*	Dose multiplier for maximum prescription dose (i.e., *γθ*_*v*_ is the maximum cumulative prescription dose for voxel *v*)
${\underline{\delta }}_{v}$	Target minimum dose for voxel *v*
${\underline{\delta }}_{v}^{i+1}$	Target minimum dose for voxel *v* for fractions *i* + 1 to *n*
$\underline{\delta }$	Vector of target minimum doses; $\underline{\delta }={\left({\underline{\delta }}_{v}\right)}_{v\in 𝓣}$
${\bar{\delta }}_{v}$	Target maximum dose for voxel *v*
${\bar{\delta }}_{v}^{i+1}$	Target maximum dose for voxel *v* for fractions *i* + 1 to *n*
$\bar{\delta }$	Vector of target maximum doses; $\bar{\delta }={\left({\bar{\delta }}_{v}\right)}_{v\in 𝓣}$
*n*	Number of fractions in the treatment
*i*	Fraction index
w*(***ℓ***, **u**)	Set of optimal solutions **w** to problem (Eq 2) when ${\underline{\delta }}_{v}=\theta_{v}$, ${\bar{\delta }}_{v}=\gamma \theta_{v}$ for each *v* ∈ 𝓣 and *P* is defined by ***ℓ*** and **u**
p*	Limiting breathing motion PMF
Δ**p** **w**	Dose distribution (vector of doses) when patient breathes according to **p** and **w** is delivered; *v*th element, for *v* ∈ 𝓥, is defined as ∑_*x* ∈ *x*_ ∑_*b* ∈ *β*_ Δ_*v*, *x*, *b*_ *p*(*x*)*w*_*b*_
**d**	Dose distribution; **d** = (*d*_*v*_)_*v* ∈ 𝓥_
**D**	Set of optimal dose distributions; **D** = {**d** ∈ ℝ^|𝓥|^ | **d** = Δp* **w** for some **w** ∈ w*(p*, p*)}
*U*(**D**, *ϵ*)	Epsilon neighborhood of **D**; *U*(**D**, *ϵ*) = {**d** ^′^ ∈ ℝ^|𝓥|^ | ‖**d** − **d** ^′^‖_1_ < *ϵ* for some **d** ∈ **D**}

### Robust optimization model with adaptation

Our framework builds on the adaptive robust framework of [1], which builds on the stylized robust model introduced in [3]. In this framework, the patient’s breathing in a given fraction is modeled by a breathing motion probability mass function (PMF) [22], which gives the frequency of each breathing motion state *x* from a finite set of breathing motion states *X*. The robust optimization model is then built around an *uncertainty set* *P*, which is a set of breathing motion PMFs that we believe may be realized in a given fraction. An uncertainty set is defined by a lower bound vector ***ℓ*** ≥ **0** and an upper bound vector **u** ≤ **1** as

$$\begin{array}{c} P=\{p\in {\mathbb{R}}^{\left|X\right|}\;|\;\forall x\in X,\;\ell \left(x\right)\leq p\left(x\right)\leq u\left(x\right);\;\sum_{x\in X}p\left(x\right)=1\}. \end{array}$$ (1)

When ***ℓ*** = **0** and **u** = **1**, *P* is the set of all PMFs on *X*, which we denote by 𝓟.

The robust optimization model is then defined as

$$\begin{array}{ccc} \text{minimize} & & \sum_{v\in 𝓥}\sum_{x\in X}\sum_{b\in 𝓑}\Delta_{v,x,b}\bar{p}\left(x\right)w_{b}\\ \text{subject}\;\text{to} & & \sum_{x\in X}\sum_{b\in 𝓑}\Delta_{v,x,b}p\left(x\right)w_{b}\geq {\underline{\delta }}_{v},\;\forall v\in 𝓣,\;p\in P,\\ & & \sum_{x\in X}\sum_{b\in 𝓑}\Delta_{v,x,b}p\left(x\right)w_{b}\leq {\bar{\delta }}_{v},\;\forall v\in 𝓣,\;p\in P,\\ & & w_{b}\geq 0,\;\forall b\in 𝓑, \end{array}$$ (2)

where all notation follows [1] (see Section 3 of that paper for further details), with the exception of ${\underline{\delta }}_{v}$ and ${\bar{\delta }}_{v}$, which respectively denote the target minimum dose and target maximum dose for each voxel *v*. We remark that the optimal solution **w** of this problem is not the beamlet intensity vector that is actually delivered; it must be scaled by the number of fractions that $\underline{\delta }$ and $\bar{\delta }$ correspond to. In particular, if we are planning for fraction *i*, and thus $\underline{\delta }$ and $\bar{\delta }$ correspond to the dose that remains to be delivered over the remaining *n* − *i* + 1 fractions, then we will scale the intensity vector **w** by 1/(*n* − *i* + 1) and deliver 1/(*n* − *i* + 1)⋅**w** to the patient in fraction *i*.

The adaptive element of our framework is the generation of a new uncertainty set from the current uncertainty set and the current breathing motion PMF, which is done by updating the lower bound vector ***ℓ*** and the upper bound vector **u** defining the uncertainty set. In this paper we use the exponential smoothing update described in [1]. This update is controlled by a parameter *α* ∈ [0, 1] that specifies how much weight is placed on the most recent PMF: lower values of *α* lead to slower rates of adaptation to new PMFs, while higher values lead to faster rates of adaptation.

### Dose reaction

The main element of our framework, which constitutes the new contribution of this paper to the existing adaptive and robust optimization framework, is dose reaction. We consider two methods for updating the target minimum vector $\underline{\delta }$ and the target maximum dose vector $\bar{\delta }$. In the *non-reactive* dose update method, we set $\underline{\delta }$ and $\bar{\delta }$ as

$$\begin{array}{c} {\underline{\delta }}_{v}^{i+1}=\frac{n-i}{n}\theta_{v}, \end{array}$$ (3)

$$\begin{array}{c} {\bar{\delta }}_{v}^{i+1}=\frac{n-i}{n}\gamma \theta_{v}, \end{array}$$ (4)

in each fraction *i* + 1, where *θ*_*v*_ is the prescribed minimum dose for voxel *v* and *γθ*_*v*_ is the prescribed maximum dose for voxel *v*, with *γ* being a constant greater than or equal to 1. In this method, the initial minimum and maximum target dose are scaled by the number of remaining fractions, and are not updated using the delivered dose.

In the *reactive* ^±^ dose update method, after observing the PMF **p** ^*i*^ in fraction *i*, we set ${\underline{\delta }}_{v}^{i+1}$ and ${\bar{\delta }}_{v}^{i+1}$ for fraction *i* + 1 as

$$\begin{array}{c} {\underline{\delta }}_{v}^{i+1}=\max\{0,\;{\underline{\delta }}_{v}^{i}-\sum_{x\in X}\sum_{b\in 𝓑}\Delta_{v,x,b}p^{i}\left(x\right)\frac{w_{b}^{i}}{n-i+1}\}, \end{array}$$ (5)

$$\begin{array}{c} {\bar{\delta }}_{v}^{i+1}=\max\{0,\;{\bar{\delta }}_{v}^{i}-\sum_{x\in X}\sum_{b\in 𝓑}\Delta_{v,x,b}p^{i}\left(x\right)\frac{w_{b}^{i}}{n-i+1}\}, \end{array}$$ (6)

for each voxel *v* ∈ 𝓣. This update reduces the target minimum and maximum dose—the “dose-to-go”—for a given tumor voxel *v* by the dose delivered to that voxel in fraction *i*; the more dose a voxel *v* has accumulated up to and including fraction *i*, the smaller ${\underline{\delta }}_{v}^{i+1}$ and ${\bar{\delta }}_{v}^{i+1}$ will be, and the less dose will be delivered to voxel *v* over the remaining *n* − *i* fractions. If a given voxel *v* has accumulated its prescribed minimum dose of *θ*_*v*_, the use of max{0, ⋅} ensures that the target minimum dose is set to zero, so that subsequent beamlet intensity vectors are not forced to deliver any dose to that voxel.

In [23] we also describe two other methods for updating $\underline{\delta }$ and $\bar{\delta }$, the *reactive* ^−^ method and the *reactive* ^+^ method. These two update methods are “one-sided” update methods: the reactive^−^ method reacts to underdose only, while the reactive^+^ method reacts to overdose only. Appropriately regarded, these methods behave similarly to reactive^±^; detailed results for them can be found in [23].

**Fig 1:**
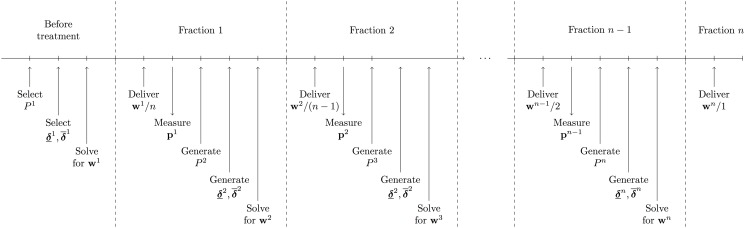
Schematic of dose-reactive method.

The complete sequence of steps involved in the dose-reactive method is displayed in Fig 1.

## Results

### Computational setup

We used the same data as in [3] and used the same computational setup as in [1]; for further details, the reader is referred to these papers. Two PMF sequences consisting of 30 PMFs were used to test the dose-reactive and non-reactive methods. The PMFs were obtained from RPM data using the method of [22]. The range of breathing motion was divided into five phases, resulting in a set *X* consisting of five breathing motion states.

Five equispaced coplanar beam directions were used, for a total of 1625 beamlets. The patient geometry was subdivided into 110,725 voxels of size 2.93 mm × 2.50 mm × 2.93 mm; the tumor voxel set 𝓣 contained all clinical target volume (CTV) voxels, which numbered 5495. The normal tissues for this patient geometry consisted of the left lung (containing the tumor), heart, esophagus and spinal cord. The dose deposition coefficients were computed from pre-treatment 4D-CT images using the method of [24]; the anatomical voxels from the CT image from one reference phase (full inhale) were transformed to corresponding voxels in other phases using voxel-based affine and non-rigid registration [25]. The dose distribution for each fraction was computed by summing the delivered beamlet intensities weighted by the Δ_*v*,*x*,*b*_ values and the realized PMF; the final treatment dose was obtained by accumulating the individual daily dose distributions.

The prescribed minimum and maximum tumor doses *θ*_*v*_ and *γθ*_*v*_ for each tumor voxel *v* were 72Gy and 79.2Gy (corresponding to *γ* = 1.1). The initial uncertainty sets used were the same as in [3]. Specifically, we considered a nominal uncertainty set (N) consisting of only the nominal PMF ($P=\{\bar{\text{p}}\}$); a margin uncertainty set (M) consisting of all PMFs (*P* = 𝓟); and a robust uncertainty set (R) that is between the nominal and margin uncertainty set in size. We also evaluated two different daily prescient treatments (see [1]). Each of the daily prescient treatments incorporates one of the two target dose updates, thus resulting in non-reactive daily prescient and reactive^±^ daily prescient treatments.

In the subsections that follow, we restrict our attention to results for the first PMF sequence, as the results obtained using the second PMF sequence were qualitatively similar. We restrict our focus to results corresponding to *α* values of 0 (no uncertainty set adaptation), 0.1 (slow adaptation), and 0.9 (fast adaptation). Complete results for both PMF sequences, as well as precise definitions of the dose metrics, can be found in [23]. To simplify the exposition, we will use the notation (⋅, ⋅) to represent each tested implementation. The first term in the parentheses indicates the method: static (S); exponential smoothing with smoothing constant *α* and the non-reactive target dose update (ES(*α*)); exponential smoothing with smoothing constant *α* and the reactive^±^ target dose update (R^±^ES(*α*)); and daily prescient with the reactive^±^ target dose update (R^±^DLYP). The second term in the parentheses, where provided, indicates the initial uncertainty set: nominal (N), robust (R) and margin (M), as defined in [3].

### Cumulative dose results

Table 2 presents dose statistics for the final dose distribution obtained from the reactive^±^ implementations. These results show that incorporating dose reaction into the adaptive robust framework leads to treatments that are acceptable with respect to the final dose distribution. With regard to the tumor dose, every reactive^±^ implementation listed in Table 2 achieves a minimum tumor dose that is within 0.5% of 72Gy. With regard to healthy tissue dose, Table 2 shows that when *α* > 0 (i.e., the uncertainty set is updated on a daily basis), all of the reactive^±^ implementations have mean left lung doses that are less than 90% and mean normal tissue doses that are less than 91% of their respective static margin treatment values.

**Table 2:** Dose statistics for the first PMF sequence under the non-reactive and reactive^±^ methods.

Implementation	Min. tumor dose		Max. tumor dose		Mean lung dose		Mean n. tissue dose	
	Gy	%^1^	Gy	%^2^	Gy	%^3^	Gy	%^4^
(R^±^ES(0),N)	71.86	99.81	79.34	100.18	17.57	86.19	9.10	89.53
(R^±^ES(0),R)	71.94	99.91	79.22	100.03	17.76	87.08	9.19	90.46
(R^±^ES(0),M)	72.00	100.00	79.20	99.99	18.57	91.06	9.50	93.54
(R^±^ES(0.1),N)	72.00	100.00	79.20	100.00	17.55	86.08	9.06	89.17
(R^±^ES(0.1),R)	72.00	100.00	79.20	100.00	17.72	86.89	9.08	89.41
(R^±^ES(0.1),M)	72.00	100.00	79.20	100.00	17.92	87.89	9.18	90.32
(R^±^ES(0.9),N)	71.99	99.99	79.21	100.01	17.58	86.22	9.06	89.13
(R^±^ES(0.9),R)	71.99	99.99	79.21	100.01	17.59	86.27	9.06	89.17
(R^±^ES(0.9),M)	71.99	99.99	79.21	100.01	17.66	86.61	9.08	89.33
(R^±^DLYP)	72.00	100.00	79.20	100.00	17.59	86.25	9.06	89.12

^1^ Percentage of the prescribed minimum dose (72Gy).

^2^ Percentage of the prescribed maximum dose (79.2Gy).

^3^ Percentage of the mean left lung dose delivered in the static margin treatment (implementation (S,M)).

^4^ Percentage of the mean normal tissue dose delivered in the static margin treatment (implementation (S,M)).

These results also indicate that the performance of the dose-reactive methods varies relative to the non-reactive methods (see Table 1 of [1] for cumulative dose results for the non-reactive static and exponential smoothing implementations). In some cases, the reactive^±^ update leads to improvement in both tumor coverage and healthy tissue dose (for example, compare (R^±^ES(0.1),R) to (ES(0.1), R)). In other cases, the performance change brought about by dose reaction is mixed. For example, comparing the (R^±^ES(0.9,M)) with the (ES(0.9),M) treatment, we can see that the mean left lung dose was improved, but the maximum tumor dose increased slightly.

**Fig 2:**
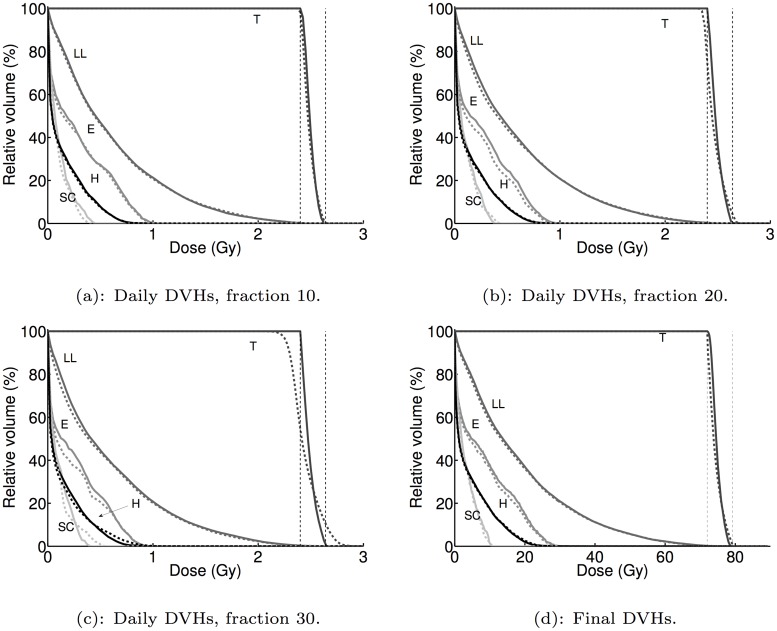
Dose-volume histograms (DVHs) for the daily dose distribution at fractions 10, 20, 30 and the final cumulative dose distribution for (ES(0.1),M) (solid curves) and (R^±^ES(0.1),M) (dashed curves) for the first PMF sequence. Letters indicate DVHs for tumor (T), left lung (LL), esophagus (E), heart (H) and spinal cord (SC).

In addition to statistics such as minimum tumor dose and mean left lung dose, we examine the full dose distribution in the patient geometry through dose volume histograms (DVHs). Fig 2(d) shows the final DVHs for the (ES(0.1),M) and (R^±^ES(0.1),M) treatments. From this figure, we see that both the (R^±^ES(0.1),M) and (ES(0.1),M) treatment result in acceptable tumor doses, while the (R^±^ES(0.1),M) treatment results in slightly lower healthy tissue dose. Overall, though, the differences between the two families of DVHs are small, indicating that in this case dose reaction has a minimal effect on the final treatment dosimetry.

### Daily dose results

**Fig 3:**
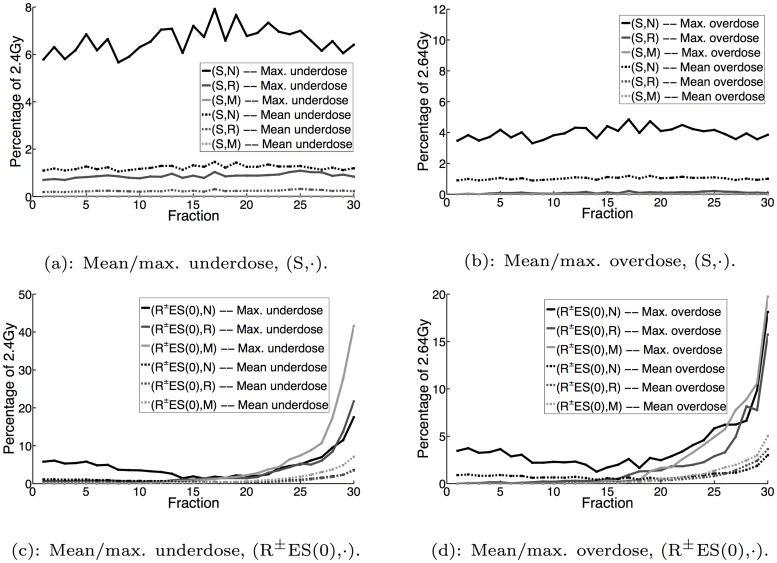
Mean and maximum underdose and overdose by fraction for S and R^±^ES(0) implementations.

**Fig 4:**
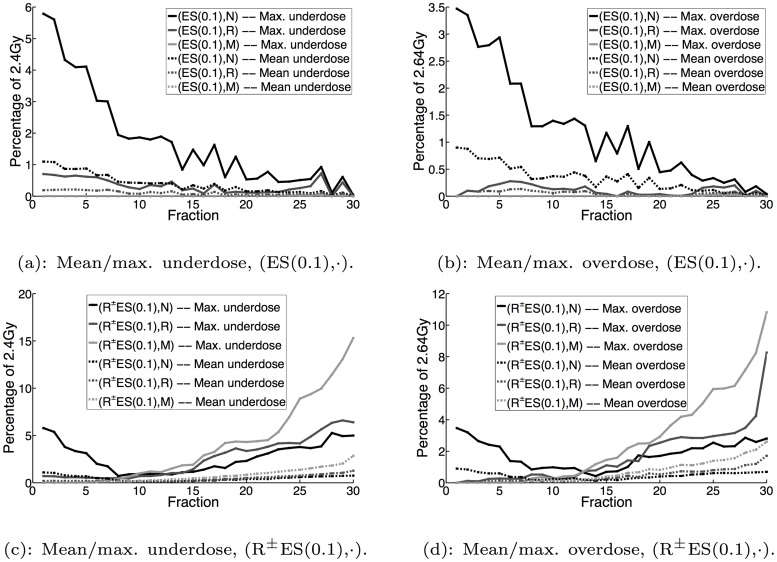
Mean and maximum underdose and overdose by fraction for ES(0.1) and R^±^ES(0.1) implementations.

**Fig 5:**
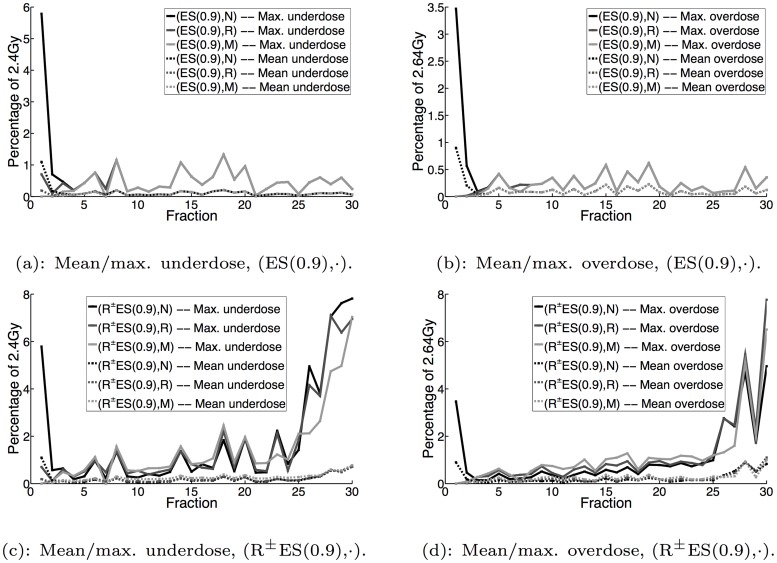
Mean and maximum underdose and overdose by fraction for ES(0.9) and R^±^ES(0.9) implementations.

**Fig 6:**
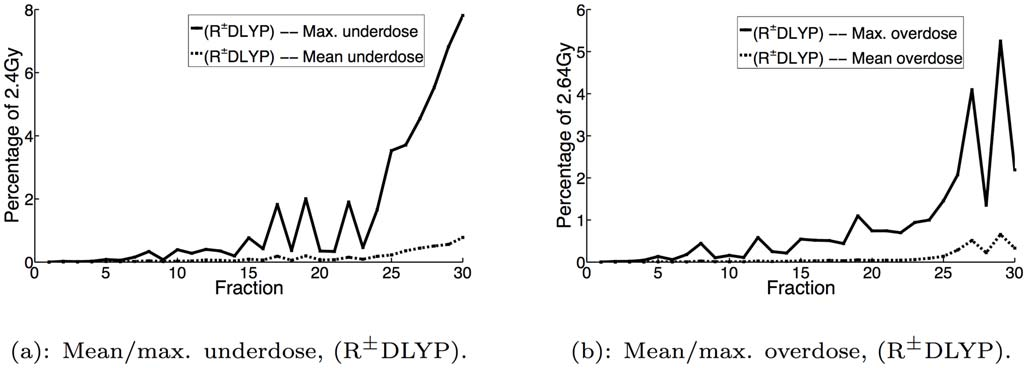
Mean and maximum underdose and overdose by fraction for R^±^DLYP implementations.

Figs 3, 4 and 5 display the mean and maximum underdose and overdose by fraction for the non-reactive and reactive^±^ implementations with *α* = 0,0.1,0.9, respectively, while Fig 6 additionally displays the same metrics for the reactive^±^ daily prescient treatment. The mean and maximum underdose values are given as percentages of the daily prescribed minimum dose of 2.4Gy (= 72Gy/30 fractions), while the mean and maximum overdose values are given as percentages of the daily prescribed maximum dose of 2.64Gy (= 79.2Gy/30 fractions). From the perspective of daily dose performance, the results shown in Figs 3, 4 and 5 indicate that the reactive^±^ method does not perform as well as the non-reactive method. In particular, we can see that the reactive^±^ method’s daily underdose and overdose grow over the course of the treatment and exceed the levels of underdose and overdose exhibited by the non-reactive method. In the non-reactive case, when *α* = 0 (the uncertainty set is not adapted), Fig 3(a) and 3(b) show that the daily underdose and overdose stay roughly constant. When *α* > 0, the daily underdose and overdose of the non-reactive method rapidly decrease to zero (e.g., see Fig 5(a) and 5(b) for *α* = 0.9).

We also examine the DVHs of the daily delivered dose distributions over the treatment course, at fraction 10 (Fig 2(a)), fraction 20 (Fig 2(b)) and fraction 30 (Fig 2(c)). At fraction 10, Fig 2(a) shows that there is little difference between (ES(0.1),M) and (R^±^ES(0.1),M). By fraction 30, however, approximately 40% of the tumor volume receives less than 2.4Gy and approximately 10% of the tumor volume receives more than 2.64Gy under (R^±^ES(0.1),M), while practically all of the tumor volume receives between 2.4Gy and 2.64Gy under (ES(0.1),M). Moreover, the tumor DVH drops from 100% to 0% at a much slower rate for (R^±^ES(0.1),M), indicating that the tumor dose for (R^±^ES(0.1),M) is less homogeneous than for (ES(0.1),M).

### Cause of daily dose performance of dose-reactive methods

Given the difference between the non-reactive and dose-reactive methods in daily dose performance that is highlighted in the previous subsection, a fundamental question arises: what causes this difference? Our results provide us with an important insight towards understanding this question. Consider Fig 6(a) and 6(b), which display the daily underdose and overdose of the daily prescient implementations. These figures provide us with an interesting insight: even if we correctly anticipate the PMF in each fraction, performing a dose-reactive update to the target dose distribution can lead to both growing daily underdose and overdose. The reason this behavior occurs in the case of the reactive^±^ daily prescient algorithms is because for a fixed PMF, it is in general not possible to deliver a completely uniform dose to the entire tumor. Therefore, in a given fraction there will be some heterogeneity in the delivered dose across the tumor, resulting in heterogeneity in the target minimum and maximum doses for the next fraction. The heterogeneity in the target dose requirements for the next fraction then leads to further heterogeneity in the dose delivered in the next fraction. As this process continues, it inevitably results in the daily dose for some voxels deviating below 2.4Gy and for some voxels deviating above 2.64Gy in the later fractions of the treatment.

**Fig 7:**
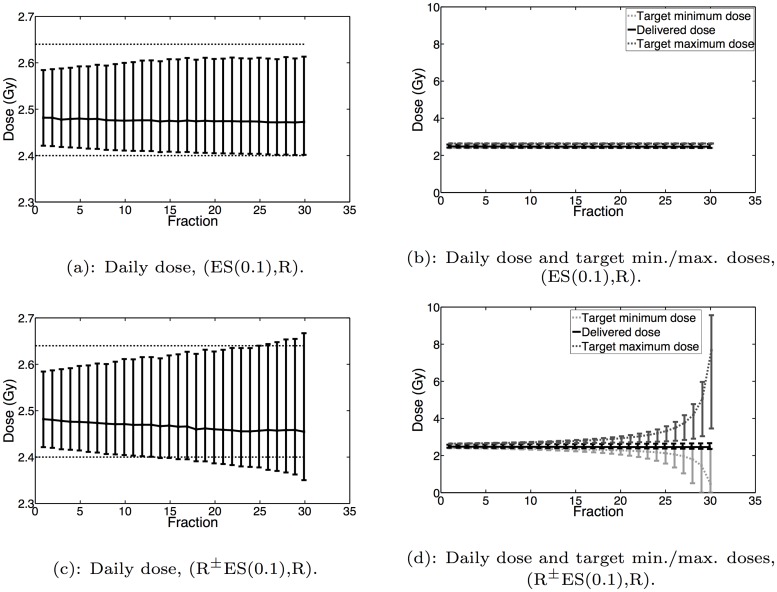
Daily delivered dose and target minimum/maximum doses for (ES(0.1),R) and (R^±^ES(0.1),R). Where shown, the lower error bar, median and upper error bar for each fraction correspond to the 5th, 50th and 95th percentiles of the appropriate distribution, respectively. On the left-hand plots, the dashed horizontal lines correspond to 2.4Gy and 2.64Gy. The target dose requirements are normalized by 1/(*n* − *i* + 1) so as to be comparable to the actual delivered dose of that fraction.

This process is further exacerbated if the prescience is removed and the uncertainty set is updated according to exponential smoothing. For these methods (R^±^ES(*α*) with *α* > 0), the daily PMF may fall outside the uncertainty set, and thus the delivered dose need not satisfy the target dose requirements. As a result, more underdose and overdose may be realized in each fraction, and the target dose requirements may grow more heterogeneous as the treatment progresses. A concrete demonstration of the growth in tumor dose heterogeneity and target minimum and maximum dose requirement heterogeneity is shown in Fig 7.

As noted in the Introduction section, the outcome of a treatment depends not only on the cumulative tumor dose delivered at the end, but also on *how* that dose was delivered to the tumor. Indeed, it has been shown that an increase in the intrafraction dose variability leads to a decrease in tumor control probability and an increase in the probability of treatment failure [6]. We have shown that dose-reactive methods generally lead to increased heterogeneity in the daily dose distribution, as well as increased daily underdose and overdose, over the non-reactive method. Therefore, a dose-reactive treatment may be more likely to fail than a non-reactive treatment, even if both treatments achieve similar final dose distributions.

### Sensitivity of daily dose performance with respect to robustness and uncertainty set adaptation

Given the growing daily underdose and overdose exhibited by dose-reactive methods, it is natural to ask how much this behavior is (1) dependent on the robust optimization component and (2) dependent on the uncertainty set adaptation component of the method. With regard to the effect of robustness, our results show that this behavior is relatively insensitive to the choice of uncertainty set. When *α* = 0 and the initial uncertainty set is the nominal uncertainty set, Fig 3(c) and 3(d) show that the daily underdose and overdose grow as the treatment progresses. This makes sense because the actual PMF in each fraction is different from the anticipated PMF, which leads to errors in dose that are compounded as the treatment progresses. However, if that singleton is replaced by the robust uncertainty set, the daily underdose and overdose still grow. This is surprising because we would expect that with a larger uncertainty set, the error between the delivered dose distribution and the target lower and upper dose bounds should be smaller, which would reduce the rate at which the daily underdose and overdose grow.

With regard to the effect of uncertainty set adaptation, our results here and in [23] show that dose-reactive implementations that adaptively update the uncertainty set (*α* > 0), generally result in slower growth in daily underdose and overdose than the dose-reactive implementations that do not modify the uncertainty set (*α* = 0); compare, for instance, Fig 3(c) and 3(d) to Fig 5(c) and 5(d). This makes sense because as the uncertainty set is updated and becomes a more accurate representation of the patient’s daily PMF, the error between the daily delivered dose distribution and the target lower and upper dose bounds should decrease, curbing how quickly the daily underdose and overdose grow. Given this last observation, our results indicate that by using dose reaction with uncertainty set adaptation—or more generally, planning the daily dose distribution with some updated estimate of the uncertainty—one can mitigate (though not fully eliminate) the growth in daily underdose and overdose.

## Theoretical insight into daily dose performance

In addition to our computational results, we advance our understanding of the daily dose performance by building on the theory developed in [1]. There, the non-reactive adaptive robust method was analyzed by assuming that the sequence of breathing motion PMFs, as an infinite sequence, converges to a limiting PMF, and evaluating the dose distribution of an *n*-fraction treatment as *n* tends to infinity. The key result in the asymptotic analysis was that for a wide class of uncertainty set update algorithms, the final cumulative dose distribution approaches a set of optimal dose distributions that exhibit no tumor underdose or overdose and low healthy tissue dose. The same theoretical framework allows us to characterize the daily performance of the non-reactive and dose-reactive methods, which we now do.

### Daily dose convergence under the non-reactive method

The following result states that the daily dose distribution of the non-reactive adaptive robust method converges to the set of optimal dose distributions. Mathematically, the result assumes that the sequence of daily PMFs converges to a limiting PMF p*. Under this assumption, the result asserts that when the lower and upper bound vectors ***ℓ*** and **u** are updated according to a *convex-convergent* update algorithm (such as exponential smoothing; see [1]), the daily dose distribution in fraction *i*, given by Δ**p** ^*i*^ **w** ^*i*^, can get arbitrarily close to a set **D** of dose distributions when *i* is sufficiently large. The set **D** is a set of optimal dose distributions that may be realized by delivering a treatment designed for *P* = {p*} when the patient breathes exactly according to p*. In a sense, the dose distributions that are realized in this way are ideal because the treatment correctly anticipates the patient’s breathing (i.e., p* ∈ *P*), and does so in the least conservative way possible (since *P* = {p*}). For further background, the reader is referred to Section 6 of [1].

**Theorem 1** Let ${\left({\text{p}}^{i}\right)}_{i=1}^{\infty }$ be a sequence of PMFs that converges to p*. Let ${\left(\ell^{i}\right)}_{i=1}^{\infty }$ and ${\left({\text{u}}^{i}\right)}_{i=1}^{\infty }$ be lower and upper bound sequences generated from ${\left({\text{p}}^{i}\right)}_{i=1}^{\infty }$ by any convex-convergent update algorithm. For each *i* ∈ ℤ_+_, let **w** ^*i*^ ∈ **w** ^⋆^(***ℓ*** ^*i*^,**u** ^*i*^). Then for every *ϵ* > 0, there exists an *N* ∈ ℤ_+_ such that for all *i* > *N*,

$$\begin{array}{c} \Delta p^{i}w^{i}\in U\left(D,\epsilon \right). \end{array}$$ (7)

**Proof**: See [23].

Note that in non-reactive treatments, the patient is treated with (1/*n*)⋅**w** ^*i*^ and not **w** ^*i*^; therefore, the actual delivered dose distribution in fraction *i* is (1/*n*)⋅Δ**p** ^*i*^ **w** ^*i*^. From Theorem 1, the actual daily delivered dose distribution (1/*n*)⋅Δ**p** ^*i*^ **w** ^*i*^ therefore tends to (1/*n*)⋅**d** for some **d** ∈ **D**, and the dose distribution (1/*n*)⋅**d** is such that each tumor voxel *v* receives exactly between *θ*_*v*_/*n* and *γθ*_*v*_/*n*. We emphasize that this result is intended as a *descriptive* result rather than a prescriptive result: it is intended to show that if the breathing motion PMFs converge or stabilize, then the daily underdose and overdose of the adaptive robust method will tend toward zero in an asymptotic sense. This statement is relevant because indeed, in Fig 4(a) and 4(b), we see that the daily underdose and overdose diminish as the treatment progresses, in agreement with the theorem.

For the dose-reactive methods, Theorem 1 does not apply. We outline here the justification; for more details, the reader is referred to [23]. The proof of Theorem 1 relies on showing that the affine functions defining the feasible region of problem (Eq 2) converge to an affine function defining the feasible region with the uncertainty set *P* = {p*}. The affine function in the *i*th fraction is dependent on the target dose requirements for the *i*th fraction. In the right-hand side plots of Fig 7 we showed how the normalized target dose requirements change as the treatment progresses for a given choice of exponential smoothing factor *α* and initial uncertainty set. From this figure, we see that the normalized target minimum and maximum dose requirements for all of the voxels do not converge to *θ*_*v*_/*n* and *γθ*_*v*_/*n* by the end of the treatment. Since these normalized target dose requirements do not necessarily converge, the affine functions defining the feasible regions of the robust problem (Eq 2) do not converge. As a result, in the limit, the dose-reactive methods can exhibit underdose or overdose in certain tumor voxels with respect to the daily prescribed minimum and maximum doses *θ*_*v*_/*n* and *γθ*_*v*_/*n*, respectively.

Furthermore, it seems unlikely that the daily dose distribution under any of the dose-reactive methods will converge under a different set of assumptions. Our computational results show that even if we consider the daily prescient treatments, which perfectly anticipate the daily PMF in every fraction, the dose-reactive methods still grow in daily underdose and overdose (cf. Fig 6(a) and 6(b)). These results give us strong reason to believe that in general, the daily dose distribution under any of the dose-reactive methods will not converge, and will not exhibit daily underdose and overdose that diminishes in the limit.

## Conclusions

In this paper, we compared the performance of methods that employ dose reaction with that of non-reactive methods using real patient data. We found that dose reaction may result in a modest improvement in both overall end-of-treatment tumor coverage and healthy tissue dose with respect to the non-reactive method. However, dose-reactive methods also result in growing daily underdose and overdose. In contrast, the non-reactive method performs well from a daily dose perspective, with daily underdose and overdose decreasing as the treatment progresses. We also proved theoretically that the daily dose distribution from the non-reactive method will converge to an ideal daily dose distribution that exhibits no tumor underdose or overdose. The same theoretical framework, together with our computational results, suggests that the dose-reactive methods will not exhibit the same convergence. The deterioration in daily dose performance we have shown has the potential to endanger the outcome of the treatment [6]. Thus, any clinical implementation of a dose-reactive strategy must account for and guard against this type of daily dose behavior to ensure a successful treatment. Overall, the key message of this paper is that reacting to dose errors—an adaptation strategy that is both simple and intuitively appealing—may backfire and lead to treatments that are clinically unacceptable.

Given the poor performance of dose-reactive methods with respect to daily dose performance, it is natural to ask how this performance can be improved. We have already highlighted the fact that uncertainty set adaptation reduces the degree of growth in daily underdose/overdose over the treatment course. An alternate avenue is to change how the dose requirements are modified. In particular, one may consider a modified dose-reactive update, where one takes a convex combination of the target minimum/maximum dose (after the normal dose-reactive update) with the corresponding non-reactive target minimum/maximum dose. More precisely, consider the following **τ*-reactive^±^* update, where *τ* ∈ [0, 1], and we set ${\underline{\delta }}_{v}^{i+1}$ for each fraction *i* + 1 as

$$\begin{array}{c} {\underline{\delta }}_{v}^{i+1}=\left(1-\tau \right)\cdot (\frac{n-i}{n})\theta_{v}+\tau \cdot \max\{0,\;{\underline{\delta }}_{v}^{i}-\sum_{x\in X}\sum_{b\in 𝓑}\Delta_{v,x,b}p^{i}\left(x\right)\frac{w_{b}^{i}}{n-i+1}\} \end{array}$$

and ${\bar{\delta }}_{v}^{i+1}$ for fraction *i* + 1 as

$$\begin{array}{c} {\bar{\delta }}_{v}^{i+1}=\left(1-\tau \right)\cdot (\frac{n-i}{n})\gamma \theta_{v}+\tau \cdot \max\{0,\;{\bar{\delta }}_{v}^{i}-\sum_{x\in X}\sum_{b\in 𝓑}\Delta_{v,x,b}p^{i}\left(x\right)\frac{w_{b}^{i}}{n-i+1}\}. \end{array}$$

In this way, the target minimum and maximum dose are not fully adjusted in response to the delivered dose and are less heterogeneous, potentially resulting in slower growth in daily underdose and overdose. Building on this idea, one may also consider a method where the constant *τ* in the formulae above is updated as the treatment progresses.
